# Rapid screening and identification of dominant B cell epitopes of HBV surface antigen by quantum dot-based fluorescence polarization assay

**DOI:** 10.1186/1556-276X-8-118

**Published:** 2013-03-02

**Authors:** Zhongji Meng, Ruihua Song, Yue Chen, Yang Zhu, Yanhui Tian, Ding Li, Daxiang Cui

**Affiliations:** 1 Center of Biotechnological Diagnosis and Therapy, The 261st Hospital of PLA, Beijing 100094, China; 2 Department of Infectious Diseases, Taihe Hospital, Hubei University of Medicine, Shiyan 442000, China; 3 Key Laboratory for Thin Film and Microfabrication Technology of Ministry of Education, Research Institute of Micro/Nano Science and Technology, Department of Bio-Nano Science and Engineering, Shanghai Jiao Tong University, Shanghai 200240, China

**Keywords:** Dominant epitope, Quantum dots, Antigenic peptide, Fluorescence polarization, HBV, Surface antigen

## Abstract

A method for quickly screening and identifying dominant B cell epitopes was developed using hepatitis B virus (HBV) surface antigen as a target. Eleven amino acid fragments from HBV surface antigen were synthesized by 9-fluorenylmethoxy carbonyl solid-phase peptide synthesis strategy, and then CdTe quantum dots were used to label the N-terminals of all peptides. After optimizing the factors for fluorescence polarization (FP) immunoassay, the antigenicities of synthetic peptides were determined by analyzing the recognition and combination of peptides and standard antibody samples. The results of FP assays confirmed that 10 of 11 synthetic peptides have distinct antigenicities. In order to screen dominant antigenic peptides, the FP assays were carried out to investigate the antibodies against the 10 synthetic peptides of HBV surface antigen respectively in 159 samples of anti-HBV surface antigen-positive antiserum. The results showed that 3 of the 10 antigenic peptides may be immunodominant because the antibodies against them existed more widely among the samples and their antibody titers were higher than those of other peptides. Using three dominant antigenic peptides, 293 serum samples were detected for HBV infection by FP assays; the results showed that the antibody-positive ratio was 51.9% and the sensitivity and specificity were 84.3% and 98.2%, respectively. In conclusion, a quantum dot-based FP assay is a very simple, rapid, and convenient method for determining immunodominant antigenic peptides and has great potential in applications such as epitope mapping, vaccine designing, or clinical disease diagnosis in the future.

## Background

An epitope or antigenic determinant is the core part of antigen involved in the recognition with an antibody. After antigens, containing numerous epitopes, are recognized by the human immunological system, B lymphocytes will synthesize and secrete miscellaneous antibodies targeting different epitopes to mediate further immunological process. Nowadays, there are many methods that are used to investigate and confirm antigen epitopes, for example, proteolytic cleavage of antigen-monoclonal antibody complexes, proteolytic or chemical cleavage fragment method, Western blotting, PEPSCAN method, chemical modification or mutation analyses, chemosynthesis of peptide, surface display of peptide libraries and random fragment expression libraries, X-ray crystallographic assay and nuclear magnetic resonance spectroscopy assay, and so on [1]. However, most of these methods are complicated, difficult to perform, or of low efficiency. With the development of computer technology and bioinformatics, a set of methods for epitope prediction were developed based on the structural property of amino acid sequences [2]; the accuracy of prediction of antigenic determinants is about 75% [3]. The methods for epitope prediction can reduce the range of possible epitope and bring us much less workloads for epitope screening. However, it is possible that some of the predicted epitopes exhibit no strong antigenicity. So, developing a novel method to analyze the antigenicity of predicted peptides has become an urgent requirement for epitope determination.

Fluorescence polarization (FP) is a unique and powerful technique for the rapid analysis of interactions of small molecular weight molecules (labeled with fluorophore) and macromolecules. The theory of fluorescence polarization was for the first time described in 1926 by Perrin [4]. When fluorescent molecules in solution are excited by a plane-polarized light beam with an appropriate wavelength, they emit fluorescent signals back into the same polarized plane, provided that the molecules remain stationary. However, if the excited molecules rotate or tumble while in the excited state, then fluorescence is emitted into a plane different from the plane used for excitation. Therefore, if the viscosity and temperature of a solution are kept constant, the degree of fluorescence polarization is dependent on the molecular volume, that is, the size of a fluorescent molecule. FP assay is based on the rotational differences between a small soluble molecule in solution (labeled with a fluorochrome) and the small molecule combined with its ligand. A small molecule can rotate randomly at a rapid rate, resulting in rapid depolarization of light, while a larger complex molecule can rotate slower and depolarize light at a reduced rate. The rate change in depolarization can be measured. High polarization values indicate that the small molecule is reacting with its ligand or target molecule, and low values mean that there is no small molecule ligand or small molecule to react with target molecule. Nowadays, homogeneous FP assays have been successfully applied to many research fields, including DNA-protein, protein-protein, and antigen-antibody binding [5-11]. However, the current FP assay is based on organic dye labeling, having some problems such as intrinsic photobleaching and low-emission efficiency, and how to solve these questions has become a great challenge.

Quantum dots have been subject to intensive investigations due to their unique properties and potential application prospect [12-14]. So far, several methods have been developed to synthesize water-soluble quantum dots (QDs) for use in biologically relevant studies [15-18]. QDs exhibit high quantum yield, high photostability, and size-dependent tunable emission, being attractive alternative luminescent labels for ultrasensitive detection and molecular imaging. For example, QDs have been used successfully in cellular imaging [19,20], immunoassays [21], DNA hybridization [22], and optical barcoding [23]. Quantum dots provide a new functional platform for bioanalytical sciences and biomedical engineering. Therefore, it is feasible to use QD labeling to improve the FP technique for detection of tumor biomarkers in patient sera [24,25].

If micromolecular antigens are adopted, FP assays can also be used to analyze the interaction of the antigen and its antibody. Herein, we reported a CdTe quantum dot-based method to screen rapidly antigenic epitopes. All possible antigenic epitopes from hepatitis B virus (HBV) surface antigen protein were predicted, and the antigenicity of peptide was determined by analyzing the recognition and combination of peptide and standard antibody samples using the FP technique. Subsequently, the immunodominant epitopes of HBV surface antigen in Chinese people with positive anti-HBV surface antigen were screened using the same method. Besides, the application of the obtained dominant antigenic peptides in detecting anti-HBV surface antibody was also investigated by FP assay.

## Methods

### Peptide sequence design

Candidate peptides were designed based on the predicted results of epitope analysis programs: the second structure of the HBV surface antigen protein sequences (UniProtiKB/Swiss-Prot: Q913A6) was predicted by the Chou-Fasman method [26], the flexible regions were analyzed by the Karplus-Schulz method [27], the hydrophilic regions were predicted by the Kyte-Doolittle method [28], the surface probability was analyzed by the Emini method [29], the antigenic index was analyzed by the Jameson-Wolf method [30], and the antigenic determinants were predicted by the Kolaskar-Tongaonkar method [3]. After comparing these multiple-parameter assay results, 11 amino acid fragments from the HBV surface antigen protein were chosen as possible epitopes. These peptides are summarized in Table 1.

**Table 1:** Designed antigenic peptide sequences from HBV surface antigen protein

**No. of peptides**	**Amino acid sequences**	**Location in HBV surface antigen protein**
1	TNLSVPNPLGFFPDHQLDP	14 to 32
2	NKVGVGA	56 to 62
3	PHGGLLGW	70 to 77
4	QAQGLLTTVPAAPP	80 to 93
5	PTPFSPPLRD	105 to 114
6	QDSRVRALYLPA	132 to 143
7	SSGTVSPAQNTVSAISSI	147 to 164
8	GGTPACPG	217 to 224
9	SQISSHSPTCCPPICPGYRW	229 to 248
10	STGPCKTCTT	291 to 300
11	MFPSCCCT	307 to 314

### Synthesis of antigenic peptides

All peptides were synthesized on 2-chlorotrityl chloride resin (1.6 mmol/g) using the standard solid-phase method of 9-fluorenylmethoxy carbonyl (Fmoc) chemistry [31]. Peptides were produced on a 0.2-mmol scale, and Fmoc-preactivated amino acids as pentafluorophenyl esters were used for the coupling reactions in the presence of hydroxybenzotriazole (Sigma Chemical Co., St. Louis, MO, USA) in dimethylformamide (DMF). Excess amino acids were used throughout the synthesis. Chain elongation reaction was performed followed by Fmoc deprotection in 20% piperidine in DMF.

When the chain elongation reaction was finished, the Fmoc protecting groups were removed from the N terminus of the peptides by 25% piperidine in DMF followed by washing with DMF for four times. Following washing for four times with DMF and dichloromethane, the resin was dried under vacuum. Subsequently, the as-prepared peptides were cleaved from the resin using standard trifluoroacetic acid (TFA) cleavage procedures in TFA with 5% H_2_O followed by multiple ether extractions. All synthetic peptides were purified to >95% by reverse-phase high-pressure liquid chromatography performed with a liquid chromatograph (Waters, Milford, MA, USA). Peptides were analyzed by mass spectrometry to confirm that the desired product was obtained.

### Preparation of QDs and QD-peptide conjugates

The CdTe QDs were prepared according to our previous report [32]. Briefly, 5 mmol of CdCl_2_·5H_2_O was dissolved in 110 mL of water, and 12 mM of thioglycolic acid (TGA) was added under stirring. NaOH solution was used to adjust the pH of the resultant solution to 11. The solution was cleared by N_2_ bubbling for 30 min. Under stirring, 2.5 mM of oxygen-free NaHTe solution was injected into the solution. After reflux at 100°C for 4 h, the TGA-capped CdTe QDs were synthesized. The obtained QDs were purified by precipitation in ethanol and redispersed in phosphate-buffered saline (PBS; 0.2 mg/mL KCl, 1.44 mg/mL Na_2_HPO_4_, 0.24 mg/mL KH_2_PO_4_, 8 mg/mL NaCl; pH 7.4). Absorbance spectrum and photoluminescence spectrum were analyzed to characterize the fluorescent properties of QDs with a PerkinElmer LS 55 spectrofluorimeter (Waltham, MA, USA).

Afterwards, 0.5 mL of 3 mg/mL QDs and 0.5 mL of 0.8 mg/mL antigenic peptides were mixed, and then 50 μL of 1 mg/mL EDC was added. The resulting solution reacted at room temperature for 3 h with continuous mixing and then stayed at 4°C for 24 h. Bovine serum albumin (BSA) was added into the solution at a concentration of 1 mg/mL and incubated at room temperature for 3 h. The QD-labeled SPAs were then centrifuged at 15,000×*g* for 30 min, and the supernatant was discarded. A volume of 1.05 mL PBS with 0.5% Tween-20 (PBST;, *v*/*v*) was used to resuspend and wash QD-labeled antigenic peptides by centrifugation at 15,000×*g* for three times. Finally, the QD-labeled conjugates were dispersed in 1.05 mL PBST and kept at 4°C for usage. Then, 1% agarose gel electrophoresis was performed to analyze the QD-peptide conjugates.

### Standard serum samples

HBV-positive sera were collected from patients who were confirmed by enzyme-linked immunosorbent assay (ELISA) test. The negative sera were collected from healthy volunteers. One hundred anti-HBV surface antigen antibody-positive sera and 100 negative sera were mixed separately at equal volume ratio. The mixtures were used as standard antibody-positive and antibody-negative serum samples.

### Fluorescence polarization assay of QD-labeled HBV antigenic peptide binding to standard antibody

Fluorescence polarization experiments were performed in a black 384-well plate (MJ Research, Waltham, MA, USA) using a Wallac Victor^2^ (1420 multilabel counter) fluorescence polarization analyzer (PerkinElmer Life Sciences). Assays were done at room temperature using filters for fluorescein excitation (480 nm) and emission (595 nm). To obtain optimal concentration for fluorescence polarization assay, QD-labeled antigenic peptides were diluted to different concentrations (from 0 to 2.5 nM, at intervals of 0.25 nM) in PBS, each of the samples was added to three wells of the 384-well plate (25 μL/well), and then the fluorescence polarization of the samples was measured. The results of the FP assay were expressed as millipolarization (mP) values, and the experiment was repeated three times.

To reduce the interference to FP values caused by impurities existing in serum samples, different dilutions (1:5, 1:10, 1:15 to 1:55) of standard serum samples were tested for FP assay. Serum samples were diluted with 2.5 nM QD-labeled peptide/PBS buffer (containing 0.2 mg/mL BSA). After thorough mixing, the mixture was added to three wells of the 384-well plate (25 μL/well) and incubated for 30 min before reading. This assay was repeated to obtain the reaction time needed for binding saturation with changed incubation time (0, 2, 5, 10, 15, 20, 25, and 30 min). The positive standard serum, negative standard serum, and diluent buffer blank control were included in the test.

According to optimal reaction factors, the antigenicity of all synthetic peptides was identified by analyzing the recognition and combination between peptides and standard antibody samples using the FP method. When the peptides bind to specific antibodies, the FP values will increase, and the increment can express the antigenicity indirectly.

### Screening for immunodominant antigenic peptides

One hundred fifty-nine samples of anti-HBV surface antigen-positive antisera were identified by the standard ELISA method with commercial ELISA kits. Specific antibodies against each peptide of HBV surface antigen with distinct antigenicity were detected using the FP method in all the antiserum samples. The distribution and levels of specific antibody against each peptide were analyzed according to the results of the FP assay.

### Detecting for HBV infection by FP assay

Using the immunodominant antigenic peptides, 293 serum samples were detected for HBV infection based on the FP assay. In order to evaluate the FP method for detection of HBV infection, ELISA experiment was carried out using a commercial ELISA kit for detection of IgG of anti-HBV. The ELISA results were used as real results; then, receiver operating characteristic (ROC) curve analysis (MedCalc Software, Ostend, Belgium) was performed on the FP assay results to determine the optimal cutoff point (at which the sum of the sensitivity and specificity values is maximal) to distinguish between positive and negative FP assay results. The ROC curve (a plot of the true-positive rate (sensitivity) against the false-positive rate (100-specificity) that is obtained at each cutoff point) was constructed, and the area under the curve (AUC) value was calculated as a measure of the accuracy of the test.

## Results and discussion

### QD conjugates and their fluorescence polarization property

**Figure 1:**
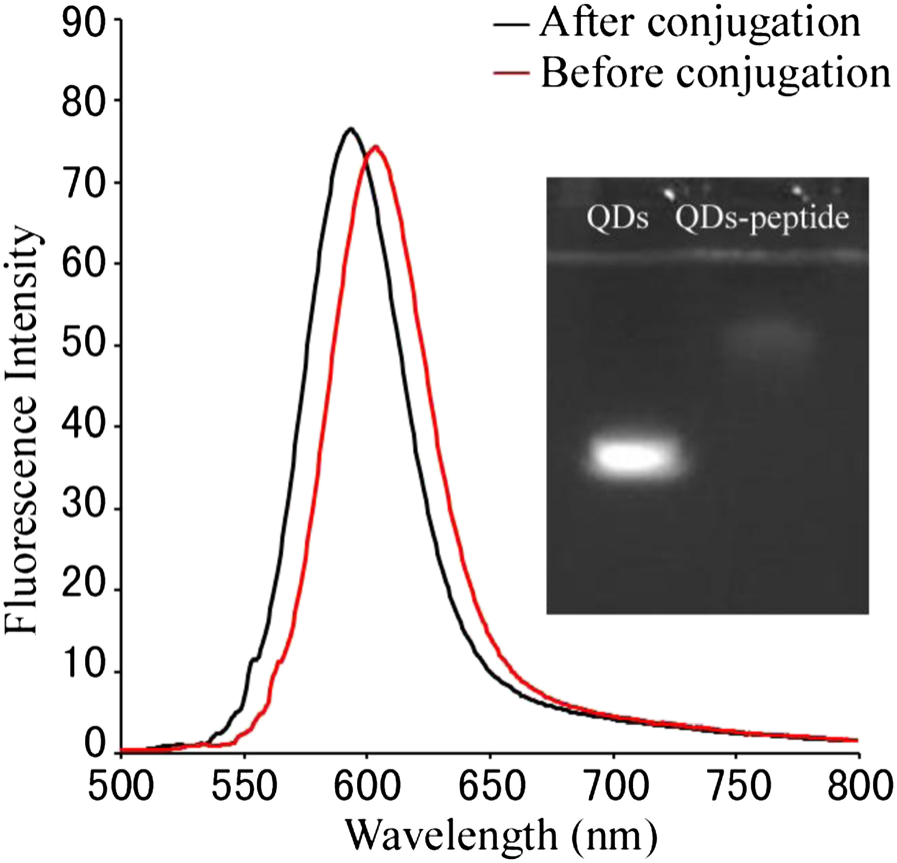
The fluorescence emission spectrum and electrophoresis of QDs and QD-peptide conjugates (inset).

**Figure 2:**
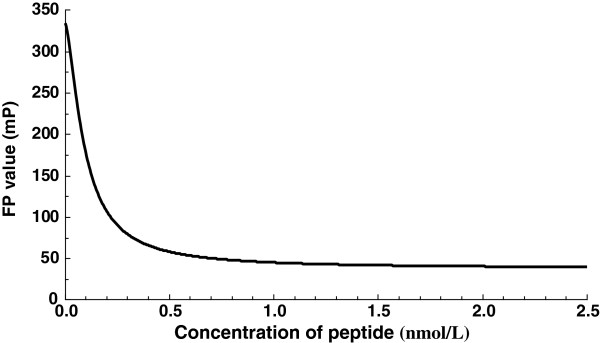
The effect of antigen concentration on FP values of QD-labeled single-epitope synthetic peptide antigen.

CdTe quantum dots were synthesized and characterized by X-ray diffraction (XRD) and high-resolution transmission electron microscopy (HR-TEM; Additional file 1: Figure S1). The QD conjugates were characterized by spectrofluorimetry and 1% agarose electrophoresis, presenting a blueshift in the maximum fluorescence wavelength and a slow electrophoretic mobility (Figure 1). The small molecular peptides labeled with QDs rotate randomly at a rapid rate in solution, resulting in rapid depolarization of light, and then, a low FP value was measured. However, the FP value increased when the concentration of fluorescent molecules was too low from our report (Figure 2). The FP value was constant only when the concentration of peptides was over 1 nmol/L.

### Dilution of serum for FP assay

**Figure 3:**
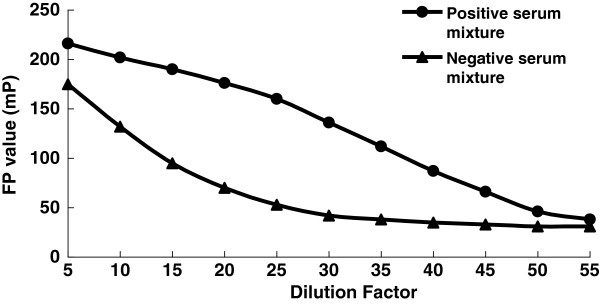
The FP values of diluted antibody-positive and antibody-negative standard serum samples.

FP value decreases when dilution times increase either for antibody-positive or for antibody-negative standard serum samples, but the downtrend for the two kinds of samples is not the same (Figure 3). These results show that there are some other molecules in the serum which can cause fluorescence polarization unexpectedly. When the dilution times are too high (>30) or too low (<20), FP values become close for antibody-positive and antibody-negative standard serum samples. The margin of FP values for the two kinds of samples reaches maximum when the serum was diluted to 25 times for FP assay.

### Incubation time for FP assay

**Figure 4:**
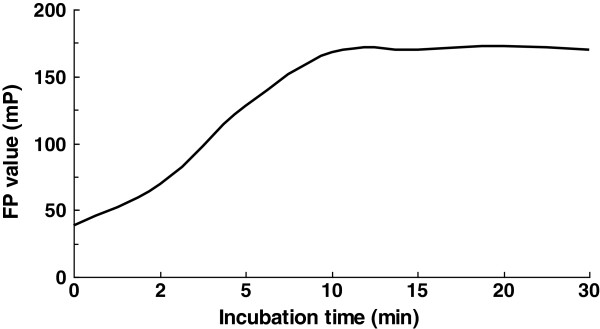
Results of FP assay at different reaction times.

The recognition and combination of peptide and standard antibody samples are very fast. The measured FP value becomes high when the peptides bind to their antibody, so the values of fluorescence polarization can represent the amount of peptide-antibody compound to some extent. FP values increase when the incubation time is prolonged to 10 min, but the FP values have no obvious change even the reaction time increases over 15 min. This shows that the reaction reaches balance after 10 to 15 min (Figure 4).

### Antigenicity of synthetic peptides

**Figure 5:**
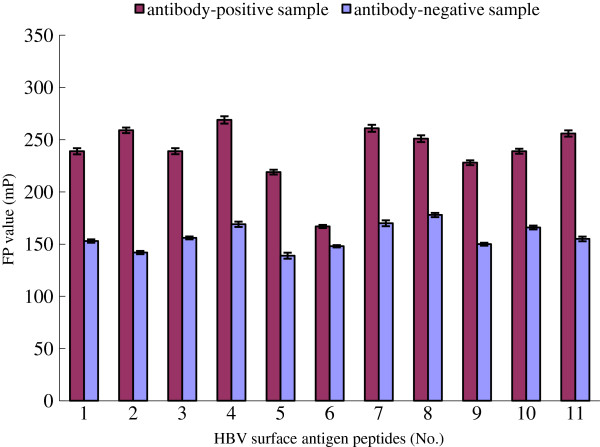
**Identification of the antigenicity of synthetic peptides by FP assay (*p *< 0.05).**

The standard antibody-positive serum sample which comprises antibodies against nearly all possible epitopes of HBV surface antigen were used to determine the antigenicity of synthetic peptides. If one peptide labeled with QDs has stronger antigenicity, more molecules of this peptide bind to its antibody in the standard serum sample; then, we can measure a higher FP value using the fluorescence polarization analyzer. On the other hand, a peptide with weaker antigenicity leads to a lower FP value. The results of FP assay show that 10 of 11 synthetic peptides (except no. 6 peptide) have antigenicity. When these 10 peptides reacted with standard antibody-positive serum, we measured >200-mP FP values, which were far higher than the FP values of those peptides that reacted with standard antibody-negative serum (Figure 5).

### Immunodominant peptides of HBV surface antigen

The dominant epitopes of HBV surface antigen were screened by analyzing the antibody levels against 10 antigenic peptides in 159 anti-HBV surface antigen-positive antiserum by FP assay. The results show that nos. 1, 10, and 11 antigenic peptides were immunodominant among 159 samples, for the antibody levels against these peptides were higher than those against other peptides or the antibodies against these peptides widely existed among 159 samples (Table 2).

**Table 2:** The results of FP assay detecting antibodies against 10 antigenic peptides in 159 serum samples

**No. of peptides**	**Numbers of samples (*n *= 159)**				**Average ΔmP**
	**ΔmP ≤ 25**	**25 < ΔmP ≤ 50**	**50 < ΔmP ≤ 100**	**ΔmP > 100**	
1	5	11	90	53	129
2	29	42	79	9	67
3	19	36	88	16	73
4	13	21	83	42	111
5	17	16	90	36	89
7	10	21	87	41	107
8	13	26	77	34	92
9	25	29	83	22	86
10	3	12	114	30	93
11	9	13	89	48	121

### Detection of HBV infection using immunodominant peptides based on FP assay

**Figure 6:**
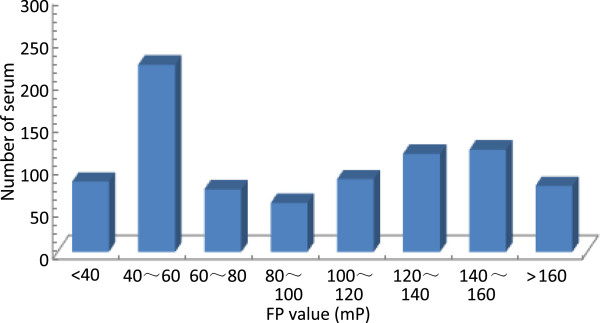
**Frequency distribution of the FP assay results that were obtained from 293 serum samples.** The *x*-axis shows the mP values, and the *y*-axis shows the number of serum.

**Figure 7:**
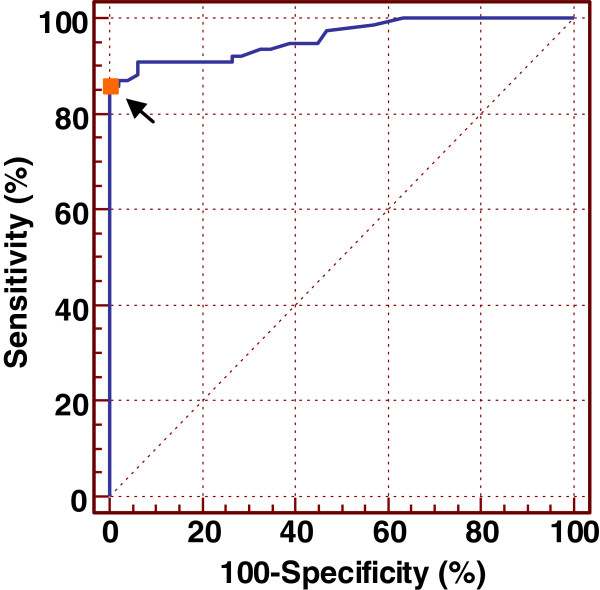
**ROC curve obtained from the analysis of the FP assay results of 293 serum samples.** The false-positive rate (100-specificity (*x*-axis)) is plotted against the true-positive rate (sensitivity (*y*-axis)) for each mP cutoff point applied. An optimal cutoff point of 77 mP is indicated (arrow). AUCROC = 0.959 (95% confidence interval = 0.908 to 0.986).

The resulting three dominant antigenic peptides were used to develop a FP-based method for detecting anti-HBV surface antigen. After FP analysis, the FP values represent the antibody levels against HBV surface antigen. The frequency distributions of the FP assay results obtained from the 293 serum samples are shown in Figure 6. The histograms show that the majority of the HBV-negative sera had mP values of <80 and the majority of the sera from infected people had mP values of ≥80. In order to distinguish positive and negative results of HBV infection by FP values, all samples were detected for HBV infection by ELISA method. The results of ELISA were used as criterion for HBV infection for each sample, and an optimal cutoff point of 77 mP for FP assay was recommended by ROC curve analysis (Figure 7). Using the FP assay method to detect HBV infection, the results indicated that the antibody-positive ratio was 51.9%, analyzed using the three antigenic peptides; the sensitivity and specificity estimates at this cutoff point were 85.4% and 98.6%, respectively. The area under the ROC curve was 0.959 (95% confidence interval = 0.908 to 0.986), which indicated a high level of accuracy for this assay.

FP assay can be used in the study of antigen-antibody interaction, and the attachment of fluorescein to antigen does not affect its ability to bind with an antibody. The only limitation of the FP assay is the size of the antigen, and the principle of FP assay restricts that only the micromolecular antigen is suitable for interaction analysis. The more differences of molecular weight between antigen and antibody exist, the more differences of FP values between free antigen and antigen-antibody complex can be measured. The molecular mass of synthetic antigenic peptides is far smaller than general antigens, so they are suitable for the screening of antigenic epitopes by the FP method. By investigating the interaction between peptide and standard antibody sample, the antigenicity of this peptide can be easily determined. For instance, when the QD-labeled peptides are mixed with the standard antibody in solution, if the peptides have antigenicity, they can bind with antibodies rapidly. The formations of antigen-antibody complex slow the rotation of the fluorescent tracer and thereby increase the polarization of the emitted light compared with only peptides existing in solution. On the other hand, the polarization has no change if the peptides have no antigenicity. In other words, a high FP value represents a strong antigenicity of peptides, and a low value represents a weak antigenicity of peptides after FP measure. When the peptides reacted with standard antibody-positive serum, in this report, the measured FP values of the 10 of 11 HBV synthetic peptides were between 200 and 250 mP, far higher than the FP values of the peptides that reacted with the standard antibody-negative serum, which were only about 150 to 170 mP, and these peptides may have antigenicity.

In order to optimize the FP assay used in detecting the interaction of antigenic peptide and antibody, we investigated the effects of different concentrations of fluorophore-labeled peptides, different dilution times of serum samples, and different incubation times of antigen-antibody mixture on FP assay. The obtained optimal factors are as follows: 1 nmol/L of fluorophore-labeled peptides, 1:25 of dilution times of serum samples, and 15 min of incubation time of antigen-antibody mixture.

The established FP assay not only can be used to identify the antigenicity of peptides with standard antibody, but can also be used to detect antibodies in serum samples with known antigenic peptides because the usages of FP assay in the two aspects share the same principle and procedures. By analyzing the antibody levels against 10 antigenic peptides in 159 anti-HBV surface antigen-positive antiserum using FP assay, we found that the antibody levels against nos. 1, 10, and 11 antigenic peptides were higher than those against other peptides or the antibodies against these peptides widely existed in 159 samples. This indicates that the three peptides may be immunodominant among 159 samples.

Based on the three dominant antigenic peptides, we also study the application of FP assay in detecting HBV infection. The FP assay data were subjected to ROC curve analysis which estimates the sensitivity and specificity of a test at every possible cutoff point and provides a measure of test accuracy. The ROC curve that was obtained from the analysis of the FP assay results indicated that the three dominant antigenic peptides are accurate indicators of HBV infection. The antibody-positive ratio was 51.9%, analyzed using the three antigenic peptides; the sensitivity and specificity estimates at the cutoff point 77 mP were 85.4% and 98.6%, respectively.

## Conclusions

In conclusion, homogeneous QD-based FP assay offers several advantages in analyzing the interaction of peptide antigen and antibody. This assay is a single-step primary binding assay using a single reagent - the QD-labeled antigenic peptides. The assay can be completed in a few minutes. Secondly, FP assay requires no repetitive washing procedures to remove unbound reactants. This also decreases the assay time considerably. In addition, the outstanding optical quality of QDs in photostability makes them an excellent fluorescent reporter. Due to the simple and rapid manipulations and high sensitivity and specificity, FP assay is very suitable for high-throughput screening of antigenic peptides and screening of immunodominant epitopes. The technical simplicity, rapid speed, and low cost of this assay make it very attractive in specific antibody detection and clinic serological tests of infectious diseases. In one word, FP assay has great applied potential in epitope mapping, vaccine designing, or clinical disease diagnosis in the future.

## Competing interests

The authors declare that they have no competing interests.

## Authors’ contributions

ZM and RS finished QD-labeling peptides and screening of antigen epitopes. YC, YZ, and YT finished identification of screened antigen epitopes. DL designed all the experiments, designed the peptides, and drafted the manuscript. DC carried out the preparation of QDs, participated in its design and coordination, and revised full manuscirpt. All authors read and approved the final manuscript.

## Supplementary Material

Additional file 1: Figure S1 Characterization of synthesized CdTe nanocrystals by XRD and HR-TEM. (A) Typical XRD patterns of prepared CdTe nanocrystals. (B) HR-TEM image shows that the synthesized CdTe nanocrystals are almost 3 nm in diameter. Click here for file
