# Supplementary material for: Rapid screening and identification of dominant B cell epitopes of HBV surface antigen by quantum dot-based fluorescence polarization assay
Source: Nanoscale Res Lett. 2013 Mar 2;8(1):118. doi: 10.1186/1556-276X-8-118 (PMC3605173; doi:10.1186/1556-276X-8-118)
Supplement: Additional file 1: Figure S1 — Characterization of synthesized CdTe nanocrystals by XRD and HR-TEM. (A) Typical XRD patterns of prepared CdTe nanocrystals. (B) HR-TEM image shows that the synthesized CdTe nanocrystals are almost 3 nm in diameter. [file 1556-276X-8-118-S1.doc]

**Supporting data**


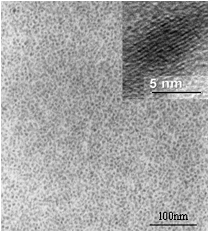


Fig s1. Characterization of synthesized CdTe nanocrystals by XRD and HR-TEM. (A) Typical XRD patterns of prepared CdTe nanocrystals. (B) HR-TEM image shows that the synthesized CdTe nanocrystals are almost 3nm in diameter.
